# Body Dissatisfaction and Self-Esteem in Female Students Aged 9–15: The Effects of Age, Family Income, Body Mass Index Levels and Dance Practice

**DOI:** 10.2478/hukin-2014-0086

**Published:** 2014-11-12

**Authors:** Lilian A. Monteiro, Jefferson S. Novaes, Mara L. Santos, Helder M. Fernandes

**Affiliations:** 1 Research Center for Sport, Health, and Human Development (CIDESD), University of Trás-os-Montes and Alto Douro (UTAD), Vila Real, PORTUGAL.; 2 Federal University of Rio de Janeiro. Physical Education - Graduate Program. Rio de Janeiro, RJ – BRAZIL.

**Keywords:** dancers, body image, public school

## Abstract

This study aimed to analyze the effects of age, family income, body mass index and dance practice on levels of body dissatisfaction and self-esteem in female students. The sample consisted of 283 female subjects attending a public school with a mean age of 11.51±1.60 years and a mean body mass index of 18.72 kg/m2 (SD=3.32). The instruments used were the Body Dissatisfaction Scale for Adolescents and the Rosenberg Self-Esteem Scale, both of which showed good internal consistency (0.77 and 0.81, respectively). The tests were applied (two-factor ANOVA) to compare the students practicing and those not practicing dance; the differences in the levels of body dissatisfaction (p=0.104) and self-esteem (p=0.09) were considered significant. The results demonstrated that age negatively correlated with body dissatisfaction (r=−0.19; p<0.01) and that higher body mass index levels were associated with greater body dissatisfaction (r=0.15, p=0.016) and lower levels of self-esteem (r=−0.17, p<0.01) only in non-practitioners. The practice of dance had a significant effect on levels of body dissatisfaction (F=4.79; p=0.030; η^2^=0.02), but there was no significant difference in self-esteem (F=1.88; p=0.172; η^2^=0.02). It can be concluded that female children and adolescents practicing dance have higher self-esteem, and are more satisfied with their body weight and their appearance. Moreover, results showed that self-esteem and body dissatisfaction were influenced by the body mass index levels only in the non-practitioners group.

## Introduction

Body dissatisfaction is understood to be an attitudinal dimension of body image and a negative evaluation that the subject makes in relation to his or her physical appearance. Life experiences create a reference for the body, and this representation is reconstructed throughout life (Forney and Ward, 2013; Spresser et al., 2012). Body image is formed from experiences that are linked to other people’s experiences, sociocultural patterns and aesthetic trends determined by the media (Esnaola et al., 2010; Schneider et al., 2013). This image is an important multidimensional construct influenced by intra and inter-individual factors and develops through personal thoughts and perceptions regarding body measurements, contours and structures (Anschutz et al., 2008; Kenyon et al., 2009).

Body dissatisfaction is a major risk factor in the development of low self-esteem, depression and behavioral disorders (Griffiths et al., 2010). Building positive self-esteem allows young people to develop positive self-efficacy and to believe they control various aspects of their lives (Scriven and Stiddard, 2003). Self-esteem can therefore be defined as the quantitative evaluative dimension of self-knowledge in terms of how an individual evaluates himself/herself. For many years, studies have treated self-esteem as a unidimensional psychological entity, regardless of the different perceptions of self that comprise it (Lindwall et al., 2011; Raustorp et al., 2005).

Body dissatisfaction decreases when physical activity is performed. This enables the individual to develop her/his body image, making the individual aware of her/his own feelings and psychophysiological reactions in relation to the body and activity and respecting its limits and possibilities (Vaquero-Cristobal et al., 2013; Varnes et al., 2013). Furthermore, the literature has demonstrated the strong influence of physical activity on building positive concepts and the formation of body self-image in young people (Campbell and Hausenblas, 2009; Gunnare et al., 2013; Kruger et al., 2008).

Dance meets these needs to develop self-references because it offers the possibility of physical activity and non-verbal communication (Huang and Gao, 2013; Murrock and Madigan, 2008; O’Neill et al., 2012). Studies have shown that dance is a tool for facilitating dancers’ interpersonal relationships and developing self-esteem, self-confidence and a sense of responsibility (Pickard and Bailey, 2009). In a mixed sample with adult dancers, relatively high levels of self-esteem were observed (Nordin-Bates et al., 2011). However, no data exist regarding possible changes in self-esteem in dancers throughout adolescence.

There is, therefore, a gap in the literature regarding the association of body dissatisfaction and self-esteem levels with the sociodemographic variables of students who are practitioners and non-practitioners of dance. Thus, this study aimed to analyze the effects of age, family income, body mass index (BMI) levels and dance practice on levels of body dissatisfaction and self-esteem in female students aged 9–15.

## Material and Methods

### Participants

The study sample was an intentional, non-probabilistic sample comprising 283 female students aged 9–15 years (M=11.51, SD=1.60) with a mean BMI of 18.72 kg/m^2^ (SD=3.32). Socioeconomic status was measured by monthly income, with 45 students (15.9%) indicating that their family received two times the minimum wage, 111 students (39.2%) reporting that their family received three times the minimum wage and the remaining 127 students (44.9%) stating that their family received four times the minimum wage. With regard to household composition, 238 students (84.1%) reported living with parents and other family members (siblings, aunt/uncle, grandparents, among others), 16 students (5.7%) reported living only with their parents, and the remaining 29 (10.2%) reported living with only their mother. With regard to skin color or race/ethnicity, the majority (n=199, 70.3%) self-reported as having brown skin color, 21.2% (n=60) as having white skin color, 8.1% (n=23) as having dark/black skin color, and 0.4% (n=1) indicated being of indigenous race/ethnicity. Regarding the practice of sports, 141 students (49.8%) were practicing dance, and 142 (50.2%) did not practice (or) had never practiced modern dance in an organized and structured way. The dance practitioners had between 12 and 96 months of dance experience (M=27.40, SD=16.31).

The procedures were carried out in accordance with the guidelines of the Declaration of Helsinki on human experimentation, and the study was approved under the protocol number 0086.0412.000-11.

### Measures

To calculate the BMI, body mass and height data were used (BMI=body mass/height^2^). BMI levels (underweight, normal weight and overweight) were defined according to the recommendations (Cole et al., 2000; Conde and Monteiro, 2006), adjusted for gender, childhood and adolescence.

The instrument used to assess body image was the Body Dissatisfaction Scale for Adolescents (Conti et al., 2009). This measure evaluates body dissatisfaction in adolescents of both genders based on information regarding the frequency of behaviors related to body care, body perception and family and social influence. It consists of 32 self-reporting questions that must be answered in accordance with six response categories: 1, never; 2, hardly ever; 3, sometimes; 4, often; 5, almost always; and 6, always. The score was calculated as follows: questions with a positive direction (questions 1–5, 7–9, 11–17, 19, 20, 22–26, 28, 30 and 31) were given a value of 0 for ‘never’, ‘hardly ever’ and ‘sometimes’ responses, a value of 1 for an ‘often’ response, a value of 2 for an ‘almost always’ response and a value of 3 for an ‘always’ response. Questions with a negative direction (questions 6, 10, 18, 21, 27, 29 and 32) had a value of 0 for an ‘always’, ‘almost always’ and ‘often’ response, a value of 1 for a ‘sometimes’ response, a value of 2 for a ‘hardly ever’ response and a value of 3 for a ‘never’ response. The score was calculated by the sum of responses and ranges from 0 to 96 points. Higher scores indicate greater body dissatisfaction of the young person. In the present study, the scale showed an internal consistency (Cronbach’s α) of 0.77.

The Rosenberg Self-Esteem Scale (1965) was also applied. This instrument has ten items, five relating to a positive view of the self and five relating to a self-deprecating view (Davis et al., 2009; Mullen et al., 2013). Response options were “disagree,” “neither agree nor disagree” and “agree”. The arrangement of the items in three-point Likert format was performed to facilitate the adolescents’ understanding. Several researchers have used modified versions of the Rosenberg Self-Esteem Scale, using a smaller or larger number of items or rewritten items as well as response options ranging between the three-and six-point Likert format according to the research objectives and the population studied (Greenberger et al., 2003; Zimprich et al., 2005). In terms of scoring, the higher the score on the scale, the higher the level of self-esteem of the subject. In this study, the coefficient of internal consistency (Cronbach’s α) was 0.81 for all scale items.

The search of schools was performed according to data provided by the Department of Education through the school registration computer system CENSO/2011, with 131 schools. The sampling criterion used was by zone, where the central zone was considered to be ten schools, of which only one had an organized dance project running, structured with social and educational activities. In the remaining schools, dance was only used in event presentations. The school boards were contacted, and they were requested to provide authorization to perform the study using the Information Statement to the Institution. Students received the Informed Consent Form to be signed by parents or guardians. Data collection was performed during class time at school from April to June 2012. The average length of time taken to complete the questionnaires and the measurements was 35 minutes.

### Statistical Analysis

The data were stored and analyzed using the SPSS 16.0 statistical software. The mean, standard deviation and percentages were calculated to provide descriptive analysis of the data. To make comparisons between the groups, analysis of variance (two-factor ANOVA) and Bonferroni *post-hoc* tests were used to identify any follow-up significance differences. The internal consistency of the scales was calculated using the Cronbach’s α. Correlation between variables was examined using the Pearson’s correlation coefficient. Significant differences and effects were set at p<0.05.

## Results

The anthropometric analysis showed that 2.5% (n=7) of the participants were underweight, 66.1% (n=187) were of normal weight, and 31.4% (n=89) were overweight (24.4% pre-obese and 7.0% obese). Analysis of the relationship between sociodemographic variables (age, family income and BMI levels) indicated that age negatively correlated with body dissatisfaction (*r*=−0.19; p<0.01) and that higher BMI levels were associated with greater body dissatisfaction (*r*=0.15; p=0.016) and lower levels of self-esteem (*r=*−0.17; p<0.01).

The table 1 presents the mean values of body dissatisfaction and self-esteem according to BMI levels.

The ANOVA results indicated that BMI levels had a significant effect on body dissatisfaction (*F*=7.25; p=0.001; η^2^=0.05) and self-esteem (*F*= 6.24; p=0.002; η^2^=0.04) scores. The Bonferroni *post-hoc* analysis indicated that children with normal weight reported lower levels of body dissatisfaction compared to those underweight (p=0.043) and those overweight (p=0.005). With regard to self-esteem, *post-hoc* analysis only revealed differences between the normal weight and overweight groups (p=0.002). Correlational analysis of the BMI with body dissatisfaction and self-esteem as a function of dance practice indicated that the BMI of non-practitioners positively correlated with body dissatisfaction (*r*=0.22; p=0.01) and negatively correlated with self-esteem (*r*=−0.030; p<0.001); however, this significant effect was not noted for the dance practitioner group. Regarding the relationship between body dissatisfaction and self-esteem, there was a similar negative correlation for the non-practitioner (*r*=−0.30; p<0.001) and practitioner (*r*=−0.31; p<0.001) groups. The analysis of variance (two-factor ANOVA) on the effect of the interaction between the practitioner group and BMI levels indicated that there was no significant interaction in levels of body dissatisfaction (p=0.104) and self-esteem (p=0.09). However, the practice of dance had a significant isolated effect on levels of body dissatisfaction (*F*=4.79; p=0.030; η^2^=0.02); there was no significant differences in the self-esteem variable (*F*=1.88; p=0.172; η^2^=0.01).

The following figures show the mean value and standard deviation for Body Dissatisfaction (1) and Self-Esteem (2) according to dance practice groups and BMI levels.

The *post-hoc* analysis of BMI levels revealed that dance practice only exerted a significant effect on overweight students, with practitioners reporting lower levels of body dissatisfaction (p=0.030) and higher levels of self-esteem (p=0.004) compared to non-practitioners.

## Discussion

This study aimed to analyze the effects of age, family income, BMI levels and dance practice on levels of body dissatisfaction and self-esteem in female students aged 9–15. The analysis of the relationship between sociodemographic variables (age, family income and BMI levels) indicated that age negatively correlated with body dissatisfaction and that higher BMI levels were associated with greater body dissatisfaction and lower levels of self-esteem. These findings suggest that students of various ages may respond differently to the requirements in relation to their body dissatisfaction and self-esteem.

In our study, children of normal weight reported lower levels of body dissatisfaction than those underweight or overweight and that there were differences between the normal weight and overweight groups with regard to self-esteem. In a study with adolescents in the city of Porto Alegre, state of Rio Grande do Sul (RS), Pinheiro and Giugliani (2006) found that most students (82%) were dissatisfied with their body and that most females, even when classified as having normal weight, were dissatisfied with their weight (58.2%). The authors warn that the youth’s body dissatisfaction can also be influenced by age, family and economic income, which can directly reflect their physical state in relation to the BMI. This was observed in our results, which indicated that higher BMI levels were associated with greater body dissatisfaction and that overweight dance practitioners expressed fewer negative feelings in relation to self-esteem than non-practitioners.

The results of the association between the BMI with body dissatisfaction and self-esteem as a function of dance practice showed that the BMI of non-practitioners positively correlated with body dissatisfaction and negatively with self-esteem; however, this significant effect was not obtained for the practitioner group. Hausenblas and Fallon (2006) also related body image to the practice of physical activity. These authors’ main results were that physically active individuals had more positive body image than sedentary ones. The authors referred to previous evidence of intervention programs in sports activities aiming to improve body image of experimental groups compared with control groups. This can be explained by the benefits that regular physical activity brings to health, providing improvement in the wellbeing of practitioners, in particular improvement in levels of self-esteem, which is considered higher in dancers than athletes in general (Nordin-Bates et al., 2011; O’Neill et al., 2012). This fact can also be observed in our results, which showed the positive effects of dance on body image satisfaction. Jordan et al. (2009) reinforced the idea that body image satisfaction was influenced not only by each person’s physical characteristics but also by comparison of these characteristics with cultural ideals and the physical appearance of their peers of the same age.

Finally, the practice of dance exerted a significant isolated effect on body dissatisfaction levels and there was no significant difference in the self-esteem variable. In several studies it has been found that physical activity helps decrease depression and improve psychological well-being, self-esteem and body image and that regardless of age and gender, physically active people have more positive self-image (Hausenblas and Fallon, 2006; Nordin-Bates et al., 2011; O’Neill et al., 2012).

Future studies should investigate the influence of these variables on other populations and on other school physical activity programs with the aim of obtaining holistic understanding of this phenomenon. Longitudinal studies may also clarify other differences and determine the cause and effect relationships between the variables investigated in the present study.

In conclusion, our results demonstrated that female children and adolescents’ practicing dance had higher self-esteem, and were more satisfied with their body mass and their appearance. Moreover, results also showed that self-esteem and body dissatisfaction were influenced by the BMI levels only in the non-practitioners group.

## Figures and Tables

**Figure 1:**
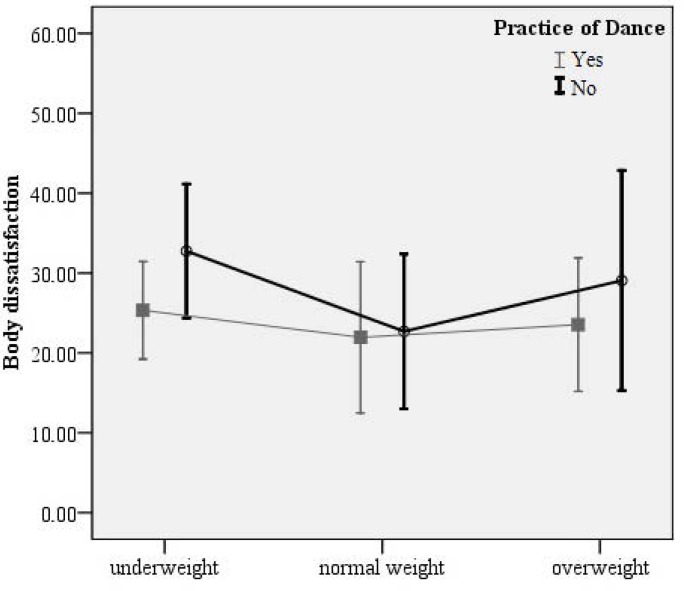
Body dissatisfaction of dance practitioners and non-practitioners according to BMI levels

**Figure 2:**
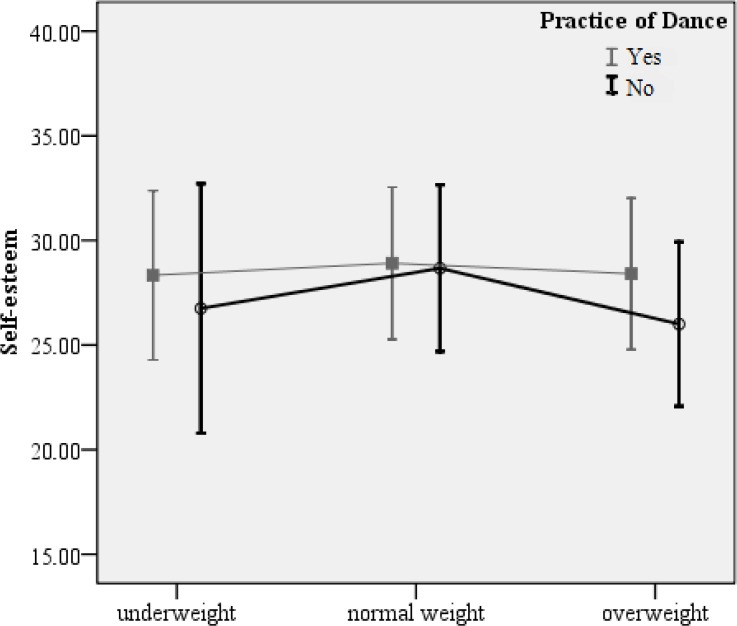
Self-esteem of dance practitioners and non-practitioners according to BMI levels

**Table 1:** The mean values of body dissatisfaction and self-esteem according to BMI levels

**Variables**	**Underweight (n=7)**	**Normal Weight (n=187)**	**Overweight (n=89)**
Body Dissatisfaction	32.43±19.99	22.30±9.56	26.64±11.97
Self-esteem	27.43±4.89	28.80±3.79	27.06±3.96

Mean values (± SD) of body dissatisfaction and self-esteem as a function of BMI levels
